# Mechanism and anti-corrosion measures of carbon dioxide corrosion in CCUS: A review

**DOI:** 10.1016/j.isci.2023.108594

**Published:** 2023-11-30

**Authors:** Ting Yan, Liang-Chen Xu, Zhuo-Xiong Zeng, Wei-Guo Pan

**Affiliations:** 1 College of Energy and Mechanical Engineering, Shanghai University of Electric Power, Shanghai 201306, China; 2 Key Laboratory of Clean Power Generation and Environmental Protection Technology in Mechanical Industry, Shanghai 200090, China

**Keywords:** Chemistry, chemical engineering, corrosion

## Abstract

Carbon capture, utilization, and storage (CCUS) technology is widely recognized as a key solution for mitigating global climate change. Consequently, it has received significant attention from countries worldwide. However, carbon dioxide corrosion poses a significant challenge to CCUS and represents a bottleneck to the large-scale development and application of this technology. To mitigate this issue, this review starts with a discussion of corrosion problems in CCUS. Later, the fundamentals of the carbon dioxide corrosion mechanism are introduced. Then, the influences of various factors that affect the corrosion are highlighted, such as water content, pH, flow rate, etc. Afterward, we summarize the commonly used methods for corrosion protection, with a particular focus on inhibitor, given their eco-friendly and effective nature. Lastly, challenges and prospects are discussed to motivate future studies on developing novel, high-performance green inhibitor and studying the corresponding protection mechanisms, hoping to make some contributions to carbon emission reduction.

## Graphical abstract

**Figure:**
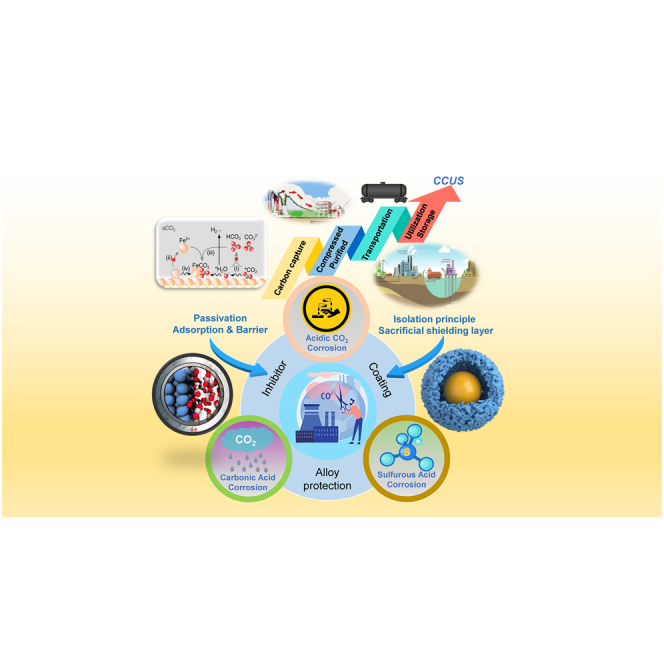

## Introduction

**Figure 1:**
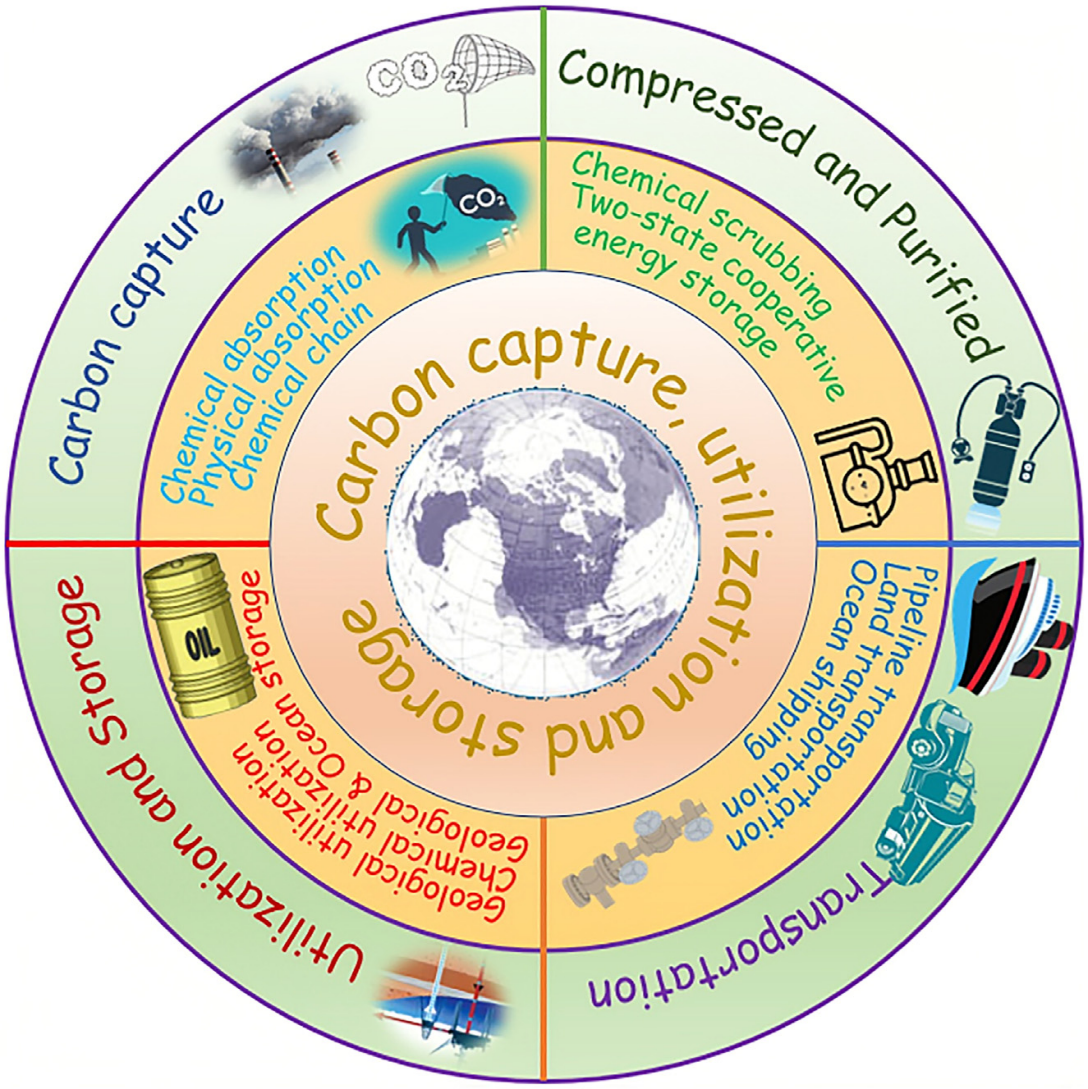
Main constituent parts of CCUS.

As the use of fossil fuels continues, CO_2_ emissions are increasing significantly. Moreover, fossil fuels remain the predominance of energy source consumption for at least in the next 30 years, although renewable energy technologies such as solar and wind energy have obtained the remarkable growth.^1^ As a result, the energy-related CO_2_ emissions will continue to increase. This trend will lead to more severe greenhouse effects, resulting in a rise in global average temperature, sea level, and rapid climate changes,^2^ which are major contributors to global warming, posing a significant threat to our planet’s health. Reducing CO_2_ emissions is crucial in mitigating the effects of climate change and protecting our planet’s fragile ecosystem. To combat this issue, the countries all over the world are vigorously advocating the implementation of various measures for energy conservation and emission reduction, with a particular focus on carbon capture, utilization, and storage (CCUS).^3^ CCUS technology is highly anticipated as a key solution in the quest for carbon neutrality.^4^ It is recognized as an effective approach to reduce CO_2_ emissions and holds the potential to significantly mitigate global warming.^5^ In fact, the related technology of CCUS has already been used for enhanced oil recovery in the oil fields for many years. The combination of CCUS and the fossil fuel power plant can achieve a clean, reliable, and sustainable energy supply. Furthermore, CCUS has brought the additional advantage of potential energy and resource recovery by employing CO_2_ as the raw material. Hence, CCUS technology is promising to serve as the foundation for constructing a zero or negative carbon emission system, presenting a prospective pathway toward mitigating carbon emissions and realizing a sustainable future.^6^^,^^7^^,^^8^ A large number of anthropogenic CO_2_ emissions mainly arise from carbon-intensive sources including fossil fuel power plants, oil refineries, cement industry, iron & steel industry, non-ferrous metals manufacturing, and building materials production, as well as the petrochemical and chemical industries.^9^ Therefore, the large-scale application of CCUS in these industries is very imperative. In the whole CCUS chain (Figure 1), CO_2_ is captured from carbon-intensive sources, compressed and purified, and then transported (usually in supercritical state) to an appropriate site for sequestration and permanent storage or to specific places for utilization in the enhanced oil recovery and converting the captured CO_2_ into valuable products.^10^ Corrosion issues tend to occur in every aspect of CCUS. Hence, the corrosion is a serious challenge in safety aspect for the whole CCUS.

**Figure 2:**
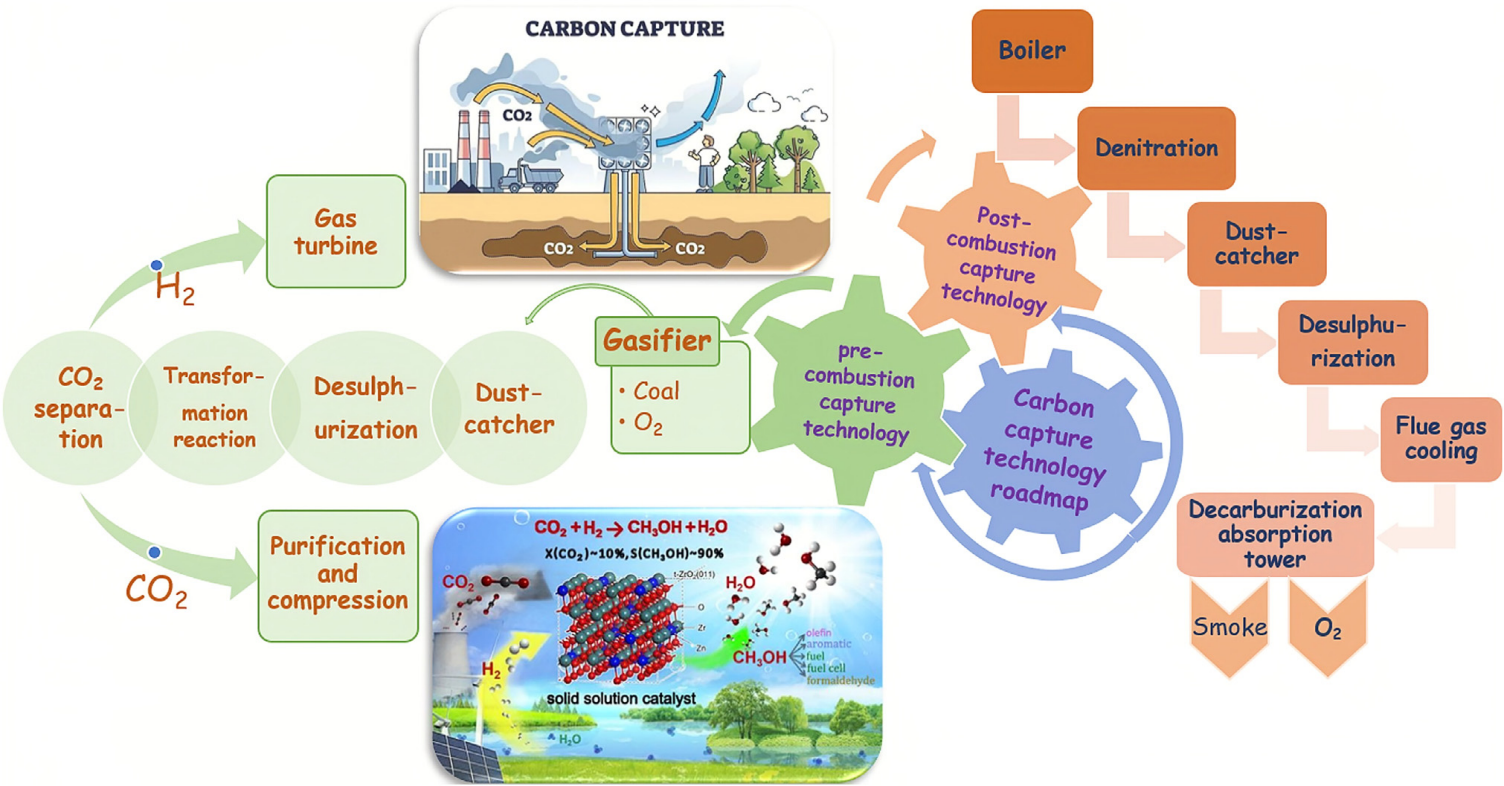
Carbon capture technology roadmap.

CO_2_ capture technology is the key part in CCUS and the most expensive and energy-intensive technical part. Currently, there are various CO_2_ capture technologies available, including physical absorption, chemical absorption, pressure swing adsorption, temperature swing adsorption, membrane separation, cryogenic separation, and others. Pre-combustion capture converts captured gas components into H_2_ and CO_2_ through chemical reaction. Post-combustion capture is to purify the flue gas first and then install a CO_2_ separation and capture device in the flue gas channel (Figure 2).^11^^,^^12^^,^^13^^,^^14^ Among various methods, the alcohol amine method is susceptible to corrode carbon steel. $\text{HC}O_{3}^{-}$, H_3_O^+^, and protonated amine are the root causes of the corrosivity of the ethanolamine solution.^15^ When CO_2_ is absorbed, the precipitated $\text{HC}O_{3}^{-}$ dissociates throughout the absorption system along with the absorption liquid, leading to overall and localized corrosion of equipment. During the capture process, any contamination or impurities can lead to corrosion.

It is worth noting that it is only a wasted resource if the collected CO_2_ cannot be efficiently and safely transported to the suitable storage locations or end use for producing the valuable chemicals. The transportation of CO_2_ can be implemented by rail, trucks, and ships (liquid phase) or via the pipeline (dense phase) in terms of the CO_2_ phase. Compared to CO_2_ capture process, the issue of CO_2_ corrosion during transportation should be even more not allowed to overlook.

Corrosion not only depletes resources but also leads to substantial economic losses. According to the reports of corrosion cost and preventive strategies in the United States, the annual direct cost incurred by corrosion is a staggering $276 billion, accounting for approximately 3.1 percent of the United States' gross domestic product in 1998.^16^ Additionally, the China Corrosion Survey Report also highlights that corrosion results in annual economic losses equivalent to approximately 5% of China’s gross domestic product. Hence, CO_2_ corrosion poses a significant challenge for the large-scale application of CCUS. However, by employing suitable material selection, corrosion monitoring, and preventive measures, and through continuous research and innovation, this issue can be effectively mitigated, ensuring the reliability and continuous operation of CCUS systems. Hence, it is crucial to investigate and analyze the formation mechanism and influencing factors.

Previous research primarily focuses on corrosion mechanism, the selection of corrosion-resistant materials, and the impact of impurities and corrosion behavior in supercritical CO_2_.^17^^,^^18^^,^^19^^,^^20^^,^^21^^,^^22^^,^^23^^,^^24^^,^^25^^,^^26^ Mubarak et al.^27^ concerned about the corrosion of oil and gas well casing and tubing and corrosion mitigation techniques. Obot et al.^28^ studied the challenges and progress of corrosion inhibitor testing under extreme conditions in the oil and gas industries. Zhang et al.^29^ discussed the superhydrophobic anti-corrosion coatings for metallic materials. Li et al.^30^ comprehensively elucidated corrosion-related behavior of alloys in supercritical CO_2_ environments. Kairy et al.^31^ reviewed corrosion of pipeline steel in dense-phase CO_2_ containing impurities. Sheetal et al.^32^ concluded corrosion inhibitor system through N-heterocyclic compounds.

The research and reviews of corrosion have largely focused on oil and gas industries, prevention measures, and corrosion-related behavior and mechanism independently, while few summarize these aspects together. In practice, understanding the mechanism can help better study how to inhibit corrosion. This work systematically reviews the recent research on corrosion in CO_2_ systems and its influencing factors, as well as anti-corrosion methods, with a particular focus on the application of corrosion inhibitor. Firstly, we give an overview of CO_2_ corrosion in CCUS. After that, corrosion mechanisms that have been widely accepted and new mechanism proposed in recent years will be provided. Plus, the research on influencing factors of CO_2_ corrosion is summarized based on recent studies. Then, the research of anti-corrosion technology in CCUS is discussed and demonstrated. Finally, the future research tendency and direction will be proposed. This study provides a thorough review of the literature to make some contribution to the action of carbon reduction. The anti-corrosion technology has a far-reaching influence and holds great potential to make breakthrough in CO_2_ reduction. The purpose of this paper is to arouse people’s attention to CO_2_ corrosion; otherwise, corrosion will become an obstacle to the large-scale application of CCUS. Looking forward to this paper can provide some salutary views for future investigations of CO_2_ corrosion mechanism and anti-corrosion measures and as well promote related scholars to achieve better improvements and extensive industrial application of CCUS.

## Carbon dioxide corrosion mechanism

To better mitigate CO_2_ corrosion, it is essential to comprehend the corrosion mechanisms associated with CO_2_. This section focuses on the corrosion mechanism of CO_2_. Here, we laid out some possible reaction mechanisms that have been put forward.

It is well known that CO_2_ gas is non-corrosive. However, once CO_2_ combines with water to form carbonic acid, it can corrode metals. CO_2_ increases the corrosion rate of metals owing to the occurrence of cathodic reaction—hydrogen evolution. The carbonic acid solution undergoes an electrochemical reaction with the metal, and the reaction process is given in the following.^33^

The overall reaction is

(Equation 1) $$\begin{array}{c} {\text{Fe}}_{\left(s\right)}+{\text{CO}}_{2\left(\text{aq}\right)}+H_{2}O_{\left(l\right)}\to {\text{Fe}}^{2+}\left(\text{aq}\right)+{{\text{CO}}_{3}}_{\left(\text{aq}\right)}+H_{2\left(g\right)} \end{array}$$

Anodic reaction is

(Equation 2) $$\begin{array}{c} {\text{Fe}}_{\left(s\right)}-2e^{-}\to {\text{Fe}}_{\left(\text{aq}\right)}^{2+} \end{array}$$

Cathodic reaction is

(Equation 3) $$2H_{\left(\text{aq}\right)}^{+}+2e^{-}\to H_{2\left(g\right)}$$

(Equation 4) $$2H_{2}{\text{CO}}_{3\left(\text{aq}\right)}+2e^{-}\to H_{2\left(g\right)}+2{\text{HCO}}_{3\left(\text{aq}\right)}^{-}$$

(Equation 5) $$2{\text{HCO}}_{3\left(\text{aq}\right)}^{-}+2e^{-}\to H_{2\left(g\right)}+2{\text{CO}}_{3\left(\text{aq}\right)}^{2-}$$

(Equation 6) $$2H_{2}O_{\left(l\right)}+2e^{-}\to H_{2\left(g\right)}+2{\text{OH}}_{\left(\text{aq}\right)}^{-}$$

**Figure 3:**
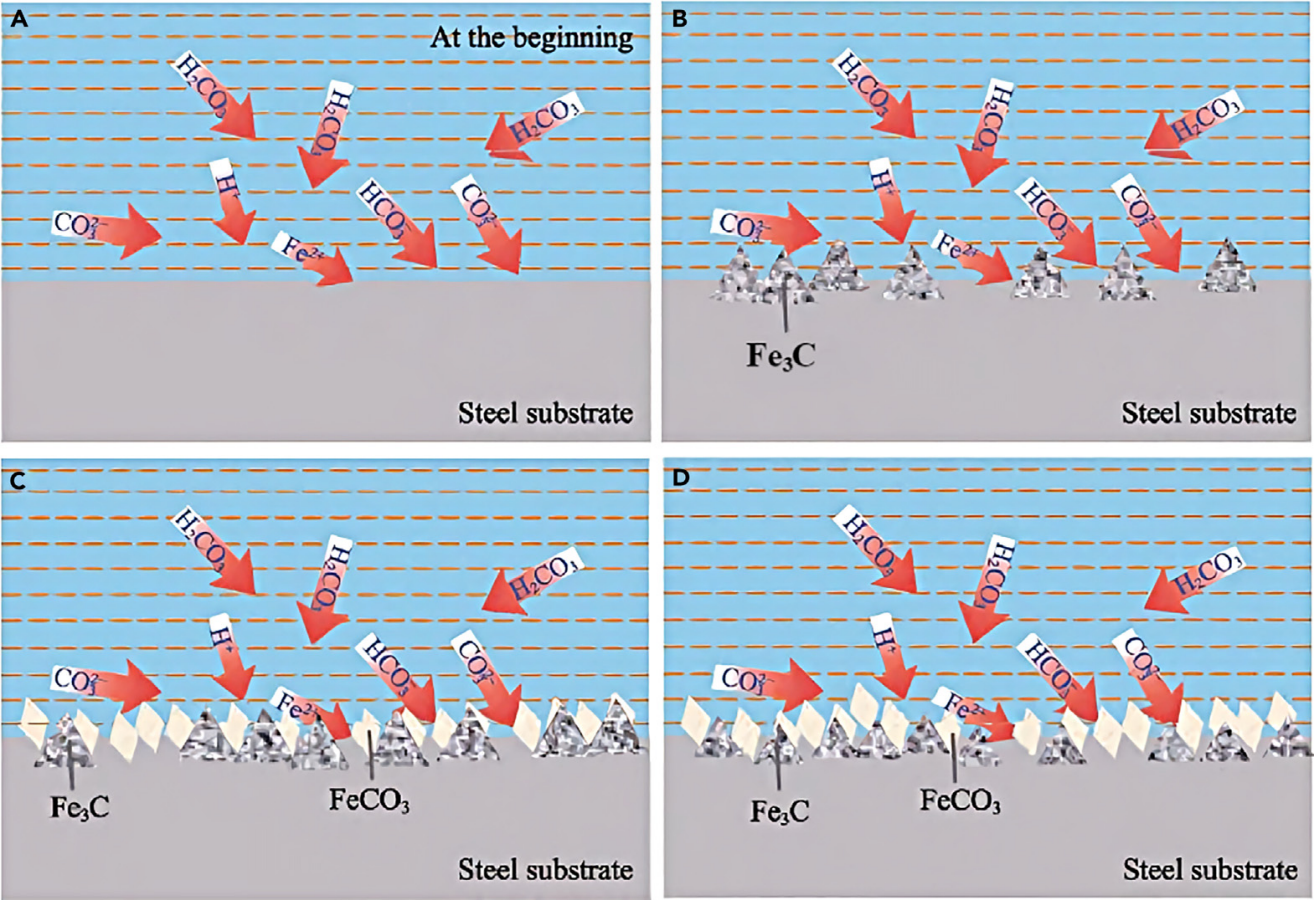
Schematic diagram of CO_2_ corrosion process on carbon steel surface.^9^

The corrosion process of CO_2_ on the metal surface in the water-rich environment involves the formation of a Fe_3_C framework within the steel substrate during the initial dissolution stage (Figure 3B), and the FeCO_3_ grains deposited on the surface (Figure 3C); then $CO_{3}^{2-}$ and $\text{HC}O_{3}^{-}$ diffused inwards and reacted with the steel matrix to form the intermediate layer and the inner layer (Figure 3D).

The layer of iron carbonate corrosion products affects most of the anodic reactions, but the cathodic reaction is not affected too much. Due to the presence of minor phases, making the corrosion product layer becomes an electronic conductor.^34^

As mentioned earlier, CO_2_ is non-corrosive, stable, and inert, and therefore CO_2_ itself does not pose a threat to metal part of CCUS. However, because of the fuels and capture technologies used for different industrial CO_2_ emitters, the collected CO_2_ including CO_2_ streams at any process of the CCUS chain always more or less contain certain number of impurities, such as H_2_O, O_2_, H_2_S, SO_2_, SO_3_, NO_2_, and organics. The existence of these impurities will induce serious corrosion problem to metal part of CCUS chain. Hence, it is very necessary to ascertain the influences of impurities on the corrosion. Moreover, the in-depth understanding of CO_2_ corrosion mechanism is a requisite for further seeking the effective protection measures of CO_2_ corrosion.

### Direct reduction of carbonic acid

Bockris et al.^35^ suggested that, under CO_2_ conditions, the intermediate medium causing Fe → Fe^2+^ (FeCO_3_) is OH^−^. Dewaard and Milliams^36^ investigated the mechanism of CO_2_ corrosion very early on. They measured the relationship between the corrosion rate of steel in carbonic acid and the partial pressure of CO_2_ and concluded that the corrosion rate may be related to the concentration of undissolved carbonic acid rather than the concentration of dissociated acid (pH). In this regard, they proposed a mechanism of cathodic reaction in line with this proposal: after initial adsorption on the metal surface, the non-dissociated carbonic acid molecules are directly reduced. The research findings of Ogundele et al.^37^ and Linter et al.^38^ closely align with the mechanism. Wiȩckowski et al.^39^ further confirmed this mechanism through the research results of cyclic voltage of static. In addition, they also observed the direct (activated) reduction of both H_2_CO_3_ molecules and $\text{HC}O_{3}^{-}$ ions.

**Figure 4:**
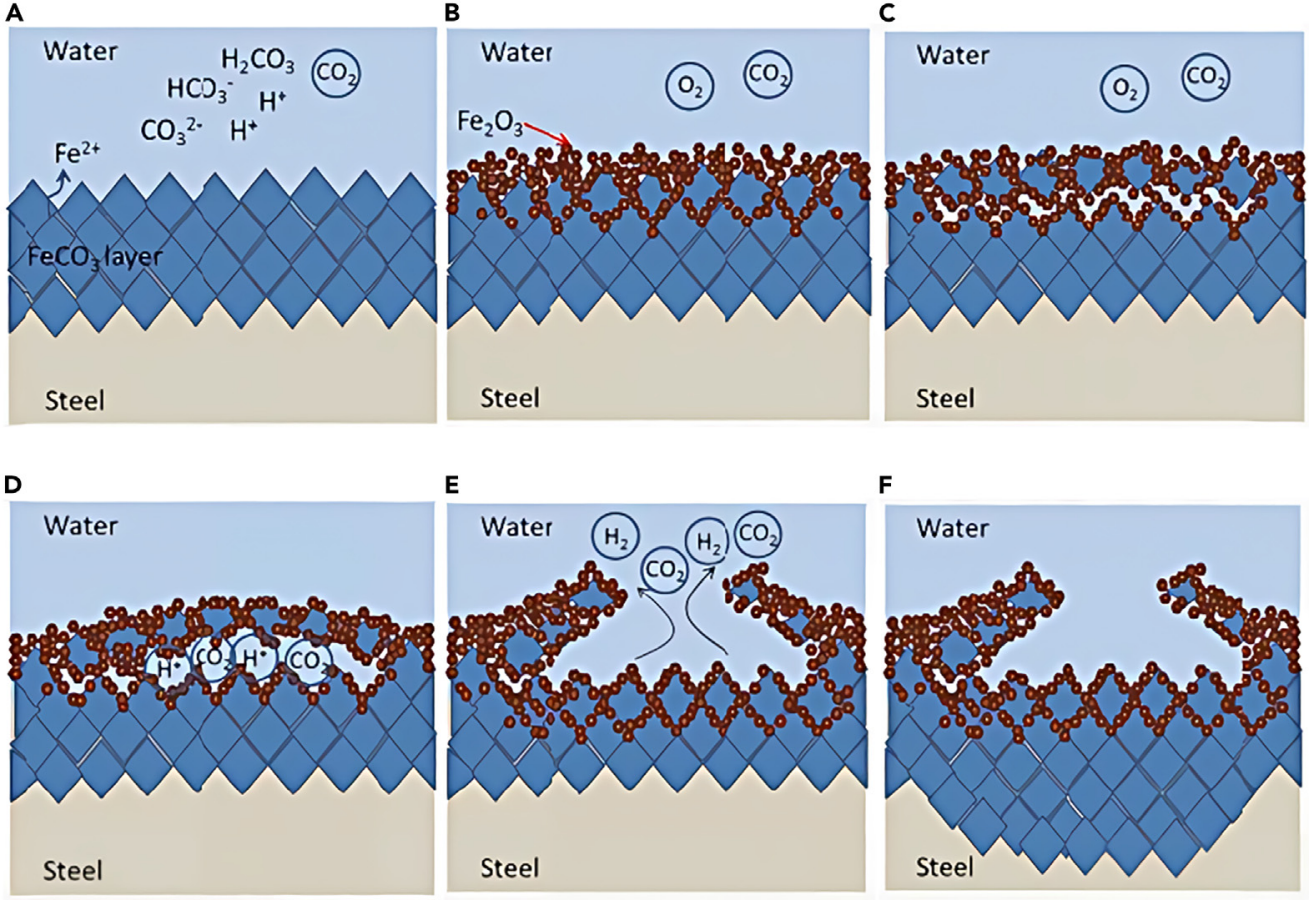
Damage mechanism of CO_2_ product film by O_2_^40^ (A) Formation of dense FeCO_3_. (B) Formation of porous Fe_2_O_3_: 4FeCO_3_+O_2_→2Fe_2_O_3_+4CO_2_. (C) O_2_ permeates and destroys FeCO_3_ layer. (D) Formation of dome-like structure. (E) Formation of oxygen concentration cell. (F) Break of thin part of dome-like film top.

For example, in environments containing CO_2_/O_2_ solution, CO_2_ combines with water to form H_2_CO_3_, which then ionizes to produce $\text{HC}O_{3}^{-}$, H^+^, and $CO_{3}^{2-}$. This leads to H^+^ reaction with the matrix Fe to generate Fe^2+^. Subsequently, Fe^2+^ and $CO_{3}^{2-}$ combine on the metal surface, resulting in the formation of a dense FeCO_3_ layer (Figure 4A). In the presence of both O_2_ and CO_2_, the metal surface becomes coated with a dense and reducible FeCO_3_ layer. Over time, FeCO_3_ gradually converts to Fe^3+^ in the presence of O_2_, thus forming porous Fe_2_O_3_ (Figure 4B). Thereafter O_2_ infiltrates into the product film, resulting in the formation of Fe_2_O_3_, which disrupts the originally dense FeCO_3_ layer on the surface (Figure 4C). During the conversion of FeCO_3_ crystal to Fe_2_O_3_, micro-pores are formed (Figure 4D). Because of the lower pH relative to the surrounding solution, metal ions undergo hydrolysis and split into H^+^ within these micro-pores. The acidified environment in the micro-pores intensifies the dissolution of FeCO_3_ and leads to the generation of CO_2_.

However, the H_2_ generated from the cathodic polarization reaction of H^+^ remains trapped in the gaps. The presence of the corrosion product film above the gaps hinders the escape of these gases, resulting in an elevated pressure within the gaps and the formation of a dome-like structure in the product film (Figure 4D). Within the micro-pore, the cathode reaction continues to consume oxygen, leading to a decrease in oxygen concentration and the establishment of an oxygen concentration cell (Figure 4E). The region with lower oxygen concentration beneath the film serves as the anode, while the area with higher oxygen concentration on the substrate surface functions as the cathode. The anode reaction leads to an increase in Fe^2+^ concentration, and the hydrolysis of Fe^2+^ further amplifies the acidity within the micro-pore. The reduced pH accelerates metal dissolution, resulting in crevice or local corrosion. The thin portion of the dome-like film on top ruptures, releasing gas and Fe^2+^ into the solution due to the continued pressure buildup within the micro-pore (Figure 4F). At this stage, the damaged film gap, along with other substrate surfaces, exhibits a galvanic effect, causing FeCO_3_ to infiltrate the active corrosion area and deposit in the micro-pore region beneath the film.

But Nesic et al.^41^ pointed out that some of the basic assumptions of the direct reduction mechanism in the previous article^35^ were problematic; because the first-order reaction of OH^−^ was reported only in the context of pH < 4, so it is not reliable for Dewaard and Milliams to directly infer higher pH. They observed different anodic mechanisms for pH < 4 and for pH > 5. In the intermediate area there seems to be a transition from one mechanism to another. Furthermore, Nesic et al.^42^ confirmed that at pH 5 the reduction of H_2_CO_3_ dominated as the cathodic reaction and summarized a reaction. This reaction is characterized by a limiting current, which is governed by a slow chemical step, namely, the hydration of CO_2_. When the current exceeds the limiting current for H^+^ or H_2_CO_3_ reduction, the primary cathodic reaction shifts to the direct reduction of water:

(Equation 7) $$\begin{array}{c} H_{2}O+e^{-}\to H+{\text{OH}}^{-} \end{array}$$

### Buffering effect

The above cathodic reaction has been widely accepted. Nevertheless, numerous scholars have put forward a new viewpoint: buffering effect.^43^^,^^44^^,^^45^^,^^46^^,^^47^ In those studies, it was quantitatively demonstrated that the limiting currents could be adequately explained even without considering H_2_CO_3_ as an electroactive species. This was elucidated by the homogeneous dissociation of H_2_CO_3_ inside the diffusion boundary layer, subsequently followed by the reduction of H^+^ that provides a parallel reaction pathway to the direct reduction of H_2_CO_3_. It becomes more comprehensible when considering the local concentration of chemical species near the metal surface. In this context, the homogeneous dissociation of H_2_CO_3_, followed by electrochemical reduction of the produced H^+^ ions, provides a parallel reaction pathway to the direct H_2_CO_3_ reduction reaction. This mechanism is not unique to CO_2_ corrosion but also exists in acetic acid corrosion of low-carbon steel.^48^ The aforementioned formula is as follows:

(Equation 8) $$H_{2}{\text{CO}}_{3}⇋{\text{HCO}}_{3}^{-}+H^{+}$$

(Equation 9) $$H^{+}+e^{-}⇋\frac{1}{2}H_{2}$$

(Equation 10) $$H_{2}\text{CO}+e^{-}⇋{\text{HCO}}_{3}^{-}+\frac{1}{2}H_{2}$$

That suggests that carbonic acid is merely a “reservoir” of hydrogen ions and its presence only increases the observed limiting current densities by buffering the H^+^ concentration at the metal surface.^43^ Carbonic acid only affects the limiting cathodic current but has no effect on the charge transfer current, and the charge transfer current only responds to changes in pH, indicating that the reduction of hydrogen ions is the primary cathodic reaction.^49^ Direct reduction mechanism is based on the assumption that H_2_CO_3_ is an electrochemical reaction material and is reduced during corrosion, while buffering mechanism emphasizes that H_2_CO_3_ is uniformly decomposed. It should be stressed that the two roles of H_2_CO_3_ (as a buffer and as an electroactive categories) are not mutually exclusive; they are two entirely different and completely independent processes.^46^ The buffering effect plays a crucial role in the CO_2_ corrosion. However, these mechanisms are not mutually exclusive, and the corrosion process of CO_2_ may encompass all of these scenarios.

## Influencing factors on carbon dioxide corrosion

CO_2_ corrosion is a complex electrochemical process influenced by a range of factors, including water content, pH, flow rate, temperature, pressure, Cl^−^ concentration, oil phase environment, and so on. Table 1 shows the influencing factors and their effects.

**Table 1:** Influencing factors and effects

Influencing factors	Effect
Water content	The corrosion rate of carbon steel materials tends to accelerate when the water content in CO_2_ increases.
pH	The higher the pH is, the lower the hydrogen ion content is, and the corrosion rate of carbon steel will also be reduced.
Flow rate	High flow rate can accelerate the rate at which the corrosion medium reaches the surface of metal pipes, potentially generating pressure that can disrupt the initially stable and compact corrosion product film, thereby increasing the corrosion rate.
Temperature	High-temperature environment can promote electrochemical reaction rates and accelerate CO_2_ corrosion.
Pressure	Within a certain pressure range, the corrosive ability of carbonated water formed by CO_2_ and formation of water gradually increases with increasing pressure.
Cl^−^	Cl^−^ has minimal impact on uniform corrosion but primarily affects localized corrosion, such as pitting corrosion.
Oil phase environment	Oil can alter the structure and chemical composition of the corrosion product film, thereby playing a role in inhibiting corrosion.

**Figure 5:**
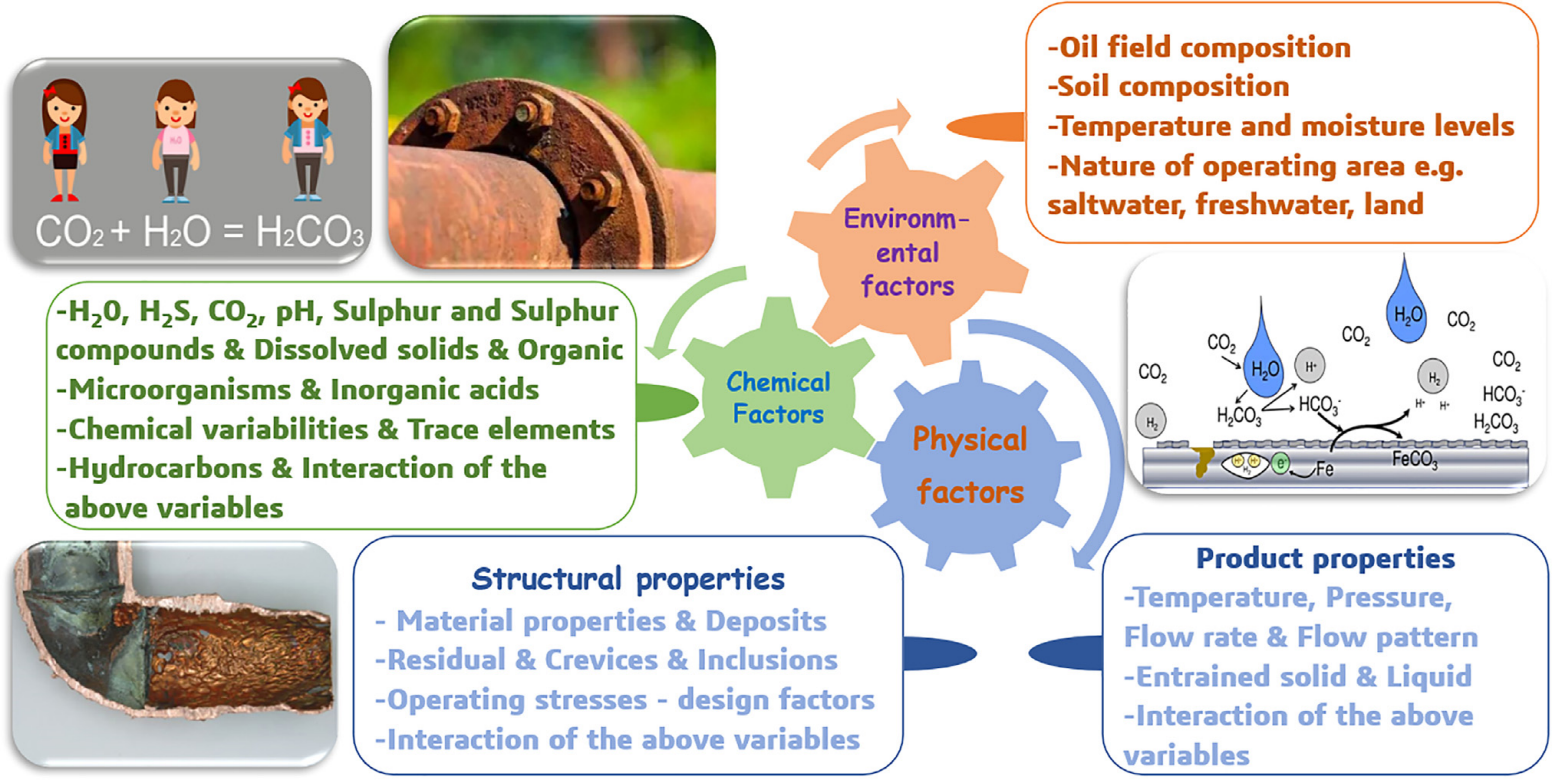
Causes of pipeline corrosion Figures reproduced from Ossai et al.^51^: Copyright 2015, Elsevier.

For instance, due to the high cost of complete purification, the captured CO_2_ fluid usually contains corrosive impurities, which can interact with CO_2_, forming more corrosive compounds and exacerbating corrosion.^50^ Pipeline corrosion can be attributed to a variety of physical and chemical factors, including environmental conditions, materials used, and more (Figure 5). Corrosion of pipeline steel often results in decreased strength and ductility, ultimately leading to pipeline ruptures or leaks. This can bring about environmental contamination and pose safety threats to personnel and equipment.^52^

Another scenario occurs during ballast voyages in cargo tanks, where the entire tank is filled with inert gas, which can often become saturated with water vapor. Throughout the voyage, this water vapor can condense and absorb any remaining sulfur-containing compounds, along with CO_2_ and nitrous oxides, giving rise to the formation of diverse acids that aggressively attack the steel, posing a threat to both the environment and the safety of the crew.^16^

### Water content

Water content is the key factor in metal corrosion. As widely recognized, carbon steel does not corrode in the presence of dry CO_2_. The variation of the corrosion rates increases with increasing relative humidity.^53^ Excessive water content will lead to electrochemical corrosion, which will seriously corrode the pipeline, shorten the service life of the pipeline, and cause economic losses. In s-CO_2_ containing with SO_2_ environment, reducing the water content is a more effective solution than reducing the sulfur dioxide content to mitigate corrosion.^54^

In s-CO_2_ transportation pipeline, the corrosion rate increases with the increase of water content, and exist the critical water content. In oil pipeline, when the water content is higher than 50%, the corrosion rate is significantly enhanced, and the corrosion form changes from uniform corrosion to local corrosion due to the uneven wetting of crude oil and water.^55^ Water solubility increases with higher temperatures and pressures within the studied range.^56^ In high water conditions with increased temperature, pressure, CO_2_ partial pressure, Ca^2+^, Cl^−^, and Mg^2+^ content, the corrosion products of 20# steel gradually increase. Consequently, the resulting corrosion product film becomes less compact, rendering it less effectively prevent corrosion.^50^ It should be noticed that the water content should be rigorously controlled when transporting CO_2_ at low temperatures and relatively low pressures.^57^ Therefore, meticulous monitoring and control of moisture content, coupled with the implementation of appropriate corrosion control measures, are of paramount importance in ensuring the long-term reliability of equipment and pipelines.

### pH

**Figure 6:**
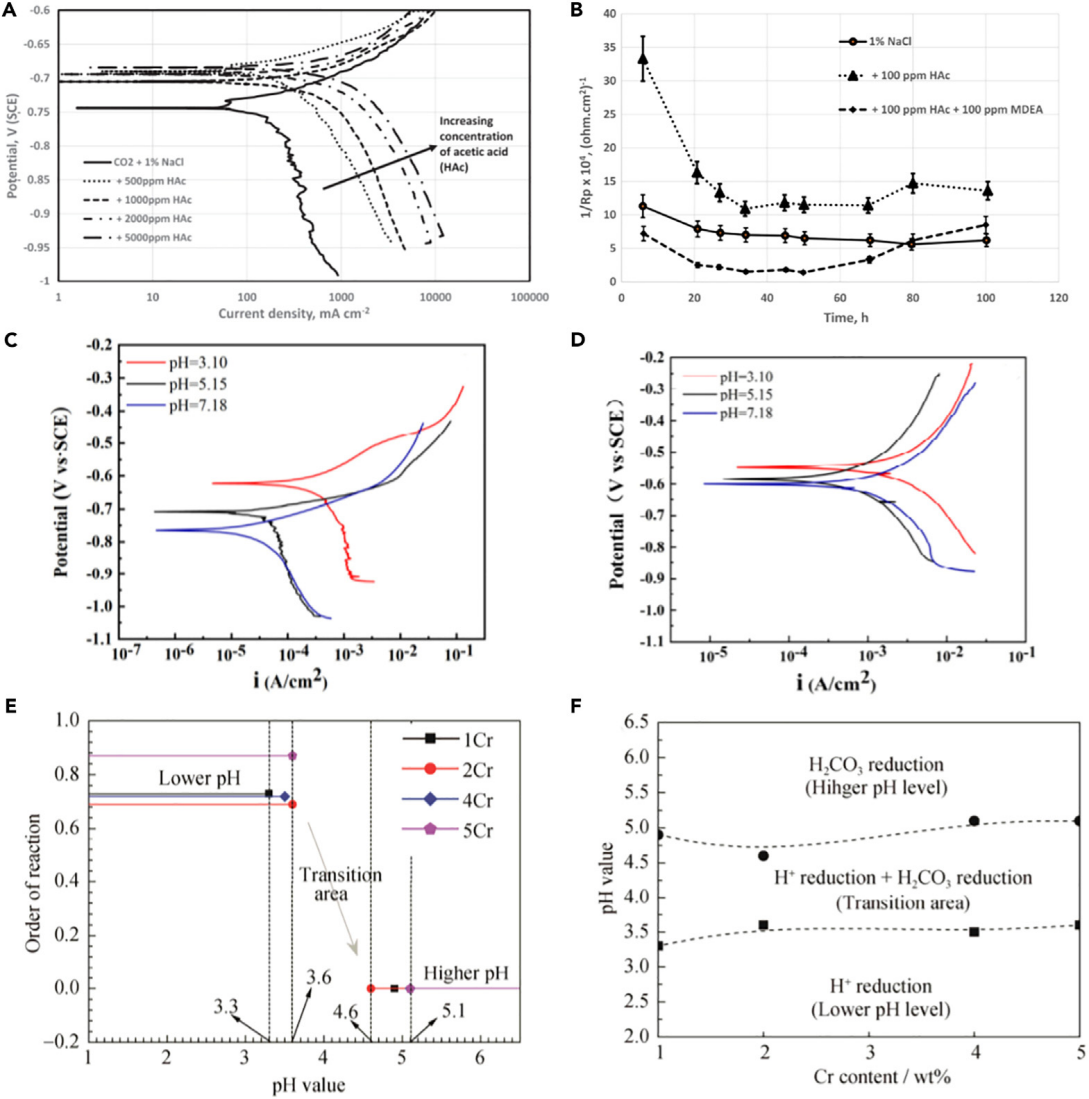
Effect of pH on corrosion (A) Potentiodynamic polarization. (B) Variation of 1/R_p_ with time. (C) Bare steel potentiodynamic polarization curves. (D) Sulfur-coated steel potentiodynamic polarization curves. (E) Order of reactions. (F) Dominant cathodic reactions. Figures reproduced from: (A and B) (Ajayi et al.^59^) Copyright 2021, John Wiley and Sons; (C and D) (Wang et al.^60^) Copyright 2021, John Wiley and Sons; (E and F) (Zhu et al.^61^) Copyright 2019, Springer Nature.

It is well known that pH will not only affect the composition of corrosion products but also affect the structure of corrosion product film, thus affecting the corrosion rate. As the pH gradually increases, it reduces the concentration of H^+^, inhibits the cathodic reaction of H^+^ depolarization during the metal corrosion process, promotes the formation of an oxidation protective film on the surface of carbon steel, and consequently reduces the corrosion rate of the metal.^58^ Based on Figures 6A and 6B, the corrosion rate can be reduced by adding methyl diethanolamine (MDEA) as it increases the pH to reduce the solubility of FeCO_3_.^59^ Additionally, lower pH will accelerate the corrosion of Fe thin films at the presence of CO_2_.^62^

In CO_2_-Cl^−^ medium with the presence of elemental sulfur, the deposition of elemental sulfur on the electrode surface can cause severe local corrosion, particularly under lower pH because the presence of sulfur and CO_2_ accelerates corrosion through direct reactions with the metal substrate or through hydrolysis to form acids.^60^ Figures 6C and 6D show the polarization curves of the bare electrode and sulfur-coated electrode at different pH levels. The presence of sulfides is more likely to induce surface cracking and unevenness.^63^ Hence, it is also essential to take into account the impact of sulfides on the corrosion process. Qin et al.^64^ and Fatah et al.^65^ also point out that sulfide will induce the generation of corrosion leading to cracking.

For low-Cr steels, the cathodic reaction is dominated by the reduction of H_2_CO_3_ and is controlled by activation when pH is greater than 5. However, at pH levels below approximately 3.5, H^+^ reduction becomes dominant and the reaction is controlled by diffusion.^61^ Figure 6E illustrates the order of reactions with respect to pH in CO_2_ corrosion of four low-Cr steels, representing the degree of influence of pH on the cathodic corrosion current. In this regard, it is suggested to ensure the stability of pH as much as possible, as fluctuations may lead to unexpected results. The dominant cathodic reactions at different pH levels are described in Figure 6F. Therefore, comprehending and controlling the pH are crucial factors in ensuring the long-term reliability and safety of equipment and pipelines.

### Flow rate

Flow rate is also an important factor affecting CO_2_ corrosion rate, for it changes the dominant corrosion type from general corrosion to local corrosion.^66^ Generally, the corrosion rate will increase with the increase of the flow rate.^67^ This is mainly because the high-speed flow accelerates the transfer of ions, which hinders the formation of corrosion product film and damages the corrosion product film already formed. The increase of flow rate increased the constant phase element of the electric double layer capacitance, reduced the charge transfer resistance, and finally accelerated the corrosion rate of steel.^68^ However, some studies showed that the corrosion rate was reduced with the increase of flow rate.^69^^,^^70^

With the increase of flow rate, the corrosion current density increases and the impedance value decreases, and the migration rate of corrosive medium on the metal surface was accelerated; thus, the formation of FeCO_3_ on the metal surface was inhibited.^58^ And the concentration of Fe^2+^ in the solution near the steel surface is reduced, the deposition of FeCO_3_ is inhibited, and then the concentration of Cr(OH)_3_ in the corrosion film is increased, thus reducing the corrosion rate.^69^

Compared with static corrosion, flowing corrosion can reduce the formation of polycrystalline layer.^71^ Nevertheless, increasing the velocity of s-CO_2_ has no significant effect on the overall/local corrosion rate under various dynamic conditions.^70^ And increasing the CO_2_ gas flow rate caused a clear reduction in the CO_2_ corrosion; since the CO_2_ gas is not as corrosive as the liquid, the corrosion rate decreases by the increased gas hold up.^72^ Simultaneously, consideration must be given to the variation of flow rate under high-temperature and high-pressure conditions. Also, it is important to care about the phenomenon of increased gas-liquid mass transfer at high flow rates. Choosing appropriate protective measures becomes crucial in mitigating the adverse effects of high flow rates on corrosion.

### Temperature

**Figure 7:**
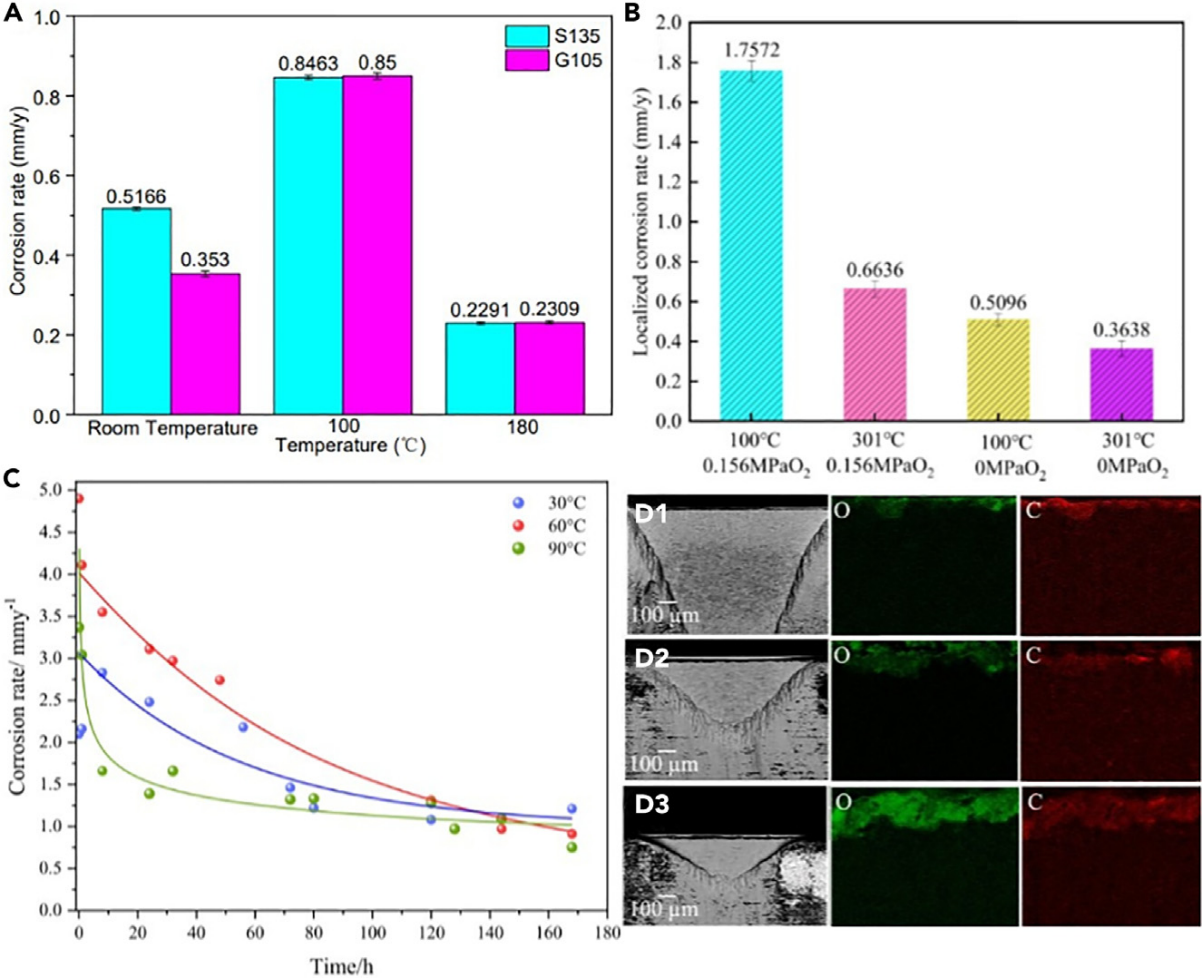
Effect of temperature on corrosion (A) Corrosion rates of S135 and G105. (B) Localized corrosion rate of 20 G steel. (C) Evolution of corrosion rates with time. (D1-D3) SEM cross-section micrographs of corrosion products. Figures reproduced from: (A) (Gao et al.^74^) Copyright 2022, MDPI; (B) (He et al.^75^) Copyright 2022, Elsevier; (C and D) (Eškinja et al.^76^) Copyright 2022, Elsevier.

Temperature affects the morphology, grain size, and density of the corrosion product film, resulting in corrosion of the substrate to varying degrees. One of the reasons elevated temperatures can exacerbate corrosion is that they lead to higher cathodic reaction kinetics.^73^ However, the corrosion rate does not necessarily increase uniformly with rising temperature. As shown in Figure 7A, the corrosion rates of S135 and G105 increase at first and then decrease with the increase of temperature. In CO_2_/H_2_S coexistence system, high temperature makes the corrosion of carbon steel worse and produces high concentration of CO_2_ and loose corrosion product film. However, with the increase of temperature, dense and uniform FeS products are formed on the surface of steel, which can inhibit corrosion.^74^

Indeed, increasing the temperature will facilitate the formation of the corrosion product film. Furthermore, in the context of a steam injection pipeline environment, the temperature’s impact on the kinetics of corrosion product film formation outweighs its influence on the thermodynamics of catalytic reactions.^75^ Due to the corrosion product film’s excellent anti-corrosive properties, this leads to a decrease in the corrosion rate with rising temperatures, as demonstrated in Figure 7B. Magdalena et al. also confirmed that higher temperatures (90°C) result in the formation of a thicker corrosion product film (FeCO_3_), which effectively reduces the corrosion rate, as depicted in Figure 7C.^76^ In addition, it is evident that increasing the temperature results in higher oxygen content and lower carbon content in the scale, which can account for the composition of the protective layer as shown in Figure 7D1-D3. The similar conclusion is also drawn by some scholars.^77^^,^^78^

Furthermore, temperature variations can also influence corrosion mechanisms. For instance, under high-temperature conditions, it may induce other corrosion mechanisms such as thermal corrosion or high-temperature hydrogen embrittlement. In summary, temperature significantly impacts the corrosion rate of CO_2_. In general, increasing temperature accelerates corrosion, but it also affects the formation of corrosion products. Its specific effects vary depending on environmental conditions and materials. Understanding the influence of temperature on corrosion aids in selecting appropriate materials and corrosion prevention measures, thereby enhancing engineering solutions.

### Pressure

**Figure 8:**
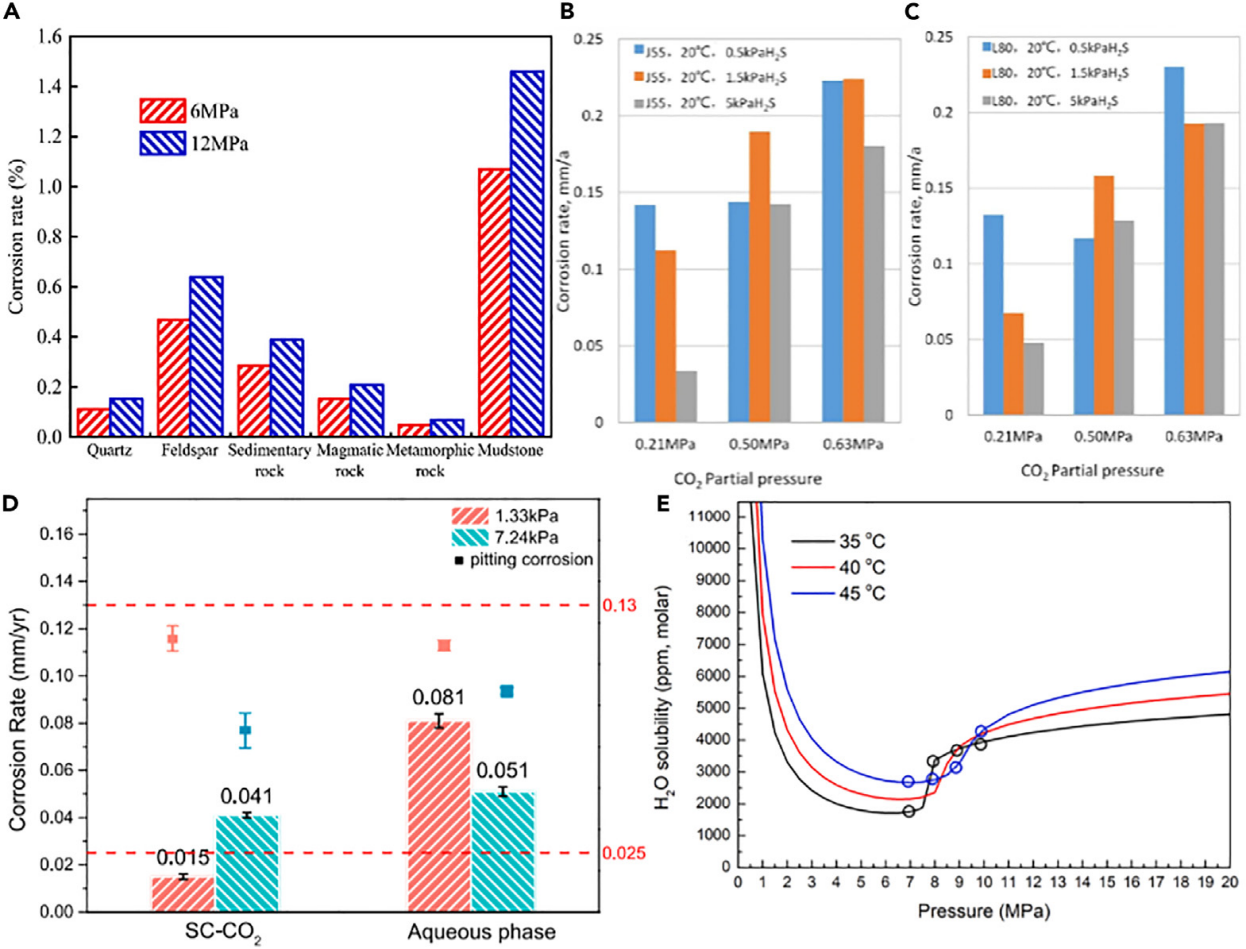
Effect of pressure on corrosion (A) Comparison of corrosion rates. (B and C) Corrosion rates of J55 and L80 steel tubes. (D) General and localized corrosion rates of sulfur-resistant steel. (E) Solubility (molar ppm) of H_2_O in CO_2_. Figures reproduced from: (A) (Tian et al.^79^) Copyright 2022, Elsevier; (B and C) (Ma^80^) Copyright 2022, Springer Nature; (D) (Wang et al.^81^) Copyright 2022, Elsevier; (E) (Li et al.^82^) Copyright 2023, Elsevier.

Pressure is one of the significant factors influencing CO_2_ corrosion, and it interacts with other factors such as temperature, CO_2_ concentration, and the type of metal. Generally speaking, under high-pressure conditions, CO_2_ is more prone to dissolve into the liquid phase, thereby increasing the concentration of CO_2_ in the corrosion medium and accelerating the corrosion reaction. As shown in Figures 8A–8D, a number of studies have reached similar conclusions.^79^^,^^80^^,^^81^ Pressure has a greater influence at a constant temperature.^83^ Additionally, localized pressure differentials can lead to the movement of gas-liquid interfaces, resulting in localized corrosion or pitting.

In s-CO_2_ phase, the corrosion was more serious with the increase of P_H2S_, while the corrosion rate of water phase decreased.^84^ There was a threshold pressure (about 10 MPa) in the transported s-CO_2_ pipeline, beyond which the corrosion rate increased significantly with the pressure.^82^ In Figure 8E, it can be observed that the solubility of H_2_O in s-CO_2_ exhibits two distinct trends, which are divided by the phase transition point. When in gaseous CO_2_, the solubility of H_2_O decreases logarithmically with increasing pressure. However, once the pressure surpasses a certain value where CO_2_ enters the liquid/supercritical state, the solubility of H_2_O increases rapidly with pressure and stabilizes at a relatively high value. Under the condition that H_2_S/CO_2_ coexist, the corrosion rate increases with the increase of H_2_S partial pressure.^84^

In the high water content period of oil-water mixed transportation pipeline, with the increase of CO_2_ partial pressure, the corrosion rate of 20# steel in the mixed pipeline gradually increases.^50^ In magnetite (Fe_3_O_4_) high-density cement-casing steel system, CO_2_ and corrosive substances tend to migrate to the cement-casing interface under high pressure, which leads to an increase in corrosion rate compared with that under normal pressure.^85^ Sun et al.^86^ also found that the corrosion potential of L80 steel shifted positively with the increase of P_CO2_. The influence of P_CO2_ on corrosion potential and current is partly attributed to the pH change of CO_2_ solution in corrosive medium. From this perspective, it becomes evident that the influence of pressure is intricately interlinked with other factors. These complex relationships need to be elucidated to gain a better understanding of the impact of pressure. This facilitates the adoption of corresponding protective measures to ensure the long-term reliability and safety of equipment and pipelines under different pressure conditions.

### Cl^−^

**Figure 9:**
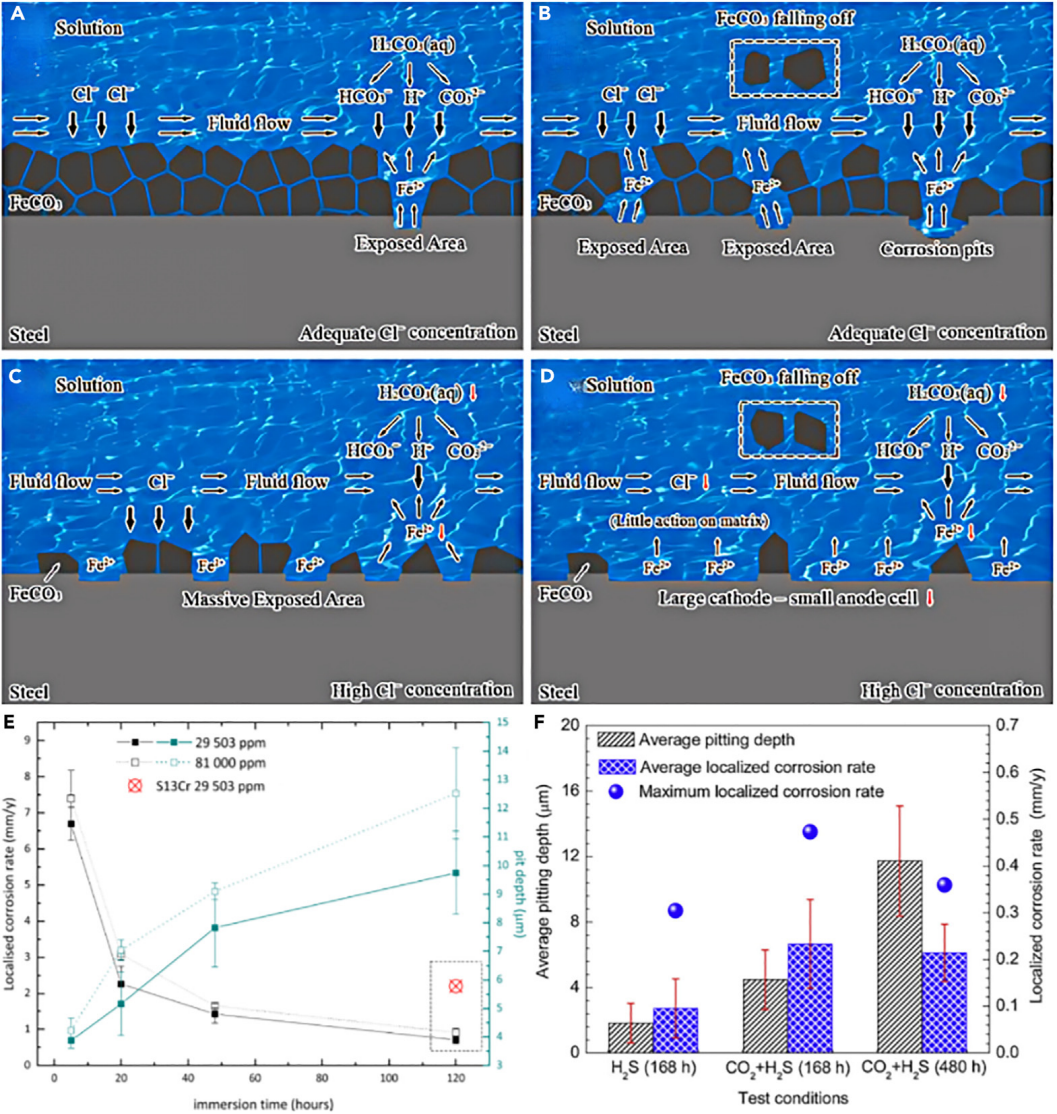
Effect of Cl^−^ on corrosion (A–D) Reverse action mechanism. (E) Localized corrosion rate and average pit depths. (F) Average pitting depth and localized corrosion rate. Figures reproduced from: (A–D) (Mou et al.^89^) Copyright 2022, Elsevier; (E) (Yue et al.^90^) Copyright 2022, Elsevier; (F) (Sun et al.^91^) Copyright 2019, Elsevier.

As one of the main anions, Cl^−^ has an aggressive effect on carbon steel. It is generally believed that Cl^−^ has little effect on uniform corrosion, while Cl^−^ mainly affects local corrosion such as pitting corrosion.^87^ The enrichment of Cl^−^ accelerates local corrosion, and the protective capacity of the passive film decreases with the increase of Cl^−^ concentration.^88^ However, Cl^−^ is not the main factor contributing to the increased corrosivity of the solution.^89^ As shown in Figures 9A–9D, the synergistic effect between adequate Cl^−^ concentration and fluid flow can promote initiation and propagation of pitting corrosion of carbon steel. In comparison, pitting behavior has been obviously limited at an extremely low surface coverage ratio induced by a combined action of an excessive Cl^−^ concentration and fluid flow.

The presence of Cl^−^ can alter the corrosion mechanism, leading to more intricate reaction pathways. For example, the increase of Cl^−^ (at low concentration) enhances the synergistic effect with O_2_, while the high content of Cl^−^ (3,000–5,000 mg/L) can reduce the solubility of CO_2_/O_2_ and replace the adsorption of corrosive media and ions on the substrate surface, thus inhibiting the cathodic process of corrosion reaction.^92^ As shown in Figure 9E, high Cl^−^ content (81,000 ppm) will lead to the precipitation of chloride-rich products, thus accelerating the general/local corrosion, inhibiting the formation of nanocrystalline NiFe_2_O_4_, and reducing the corrosion resistance.^90^

In addition, because the radius of Cl is very small, it can penetrate into the metal surface through the defects in the corrosion layer and accumulate in the pits of the iron oxide layer.^93^ The chemical reaction formula of Cl^−^ and Fe ions are as follows:

(Equation 13) $$\text{Fe}+{\text{Cl}}^{-}+H_{2}O\to {\left[\text{FeCl}\left(\text{OH}\right)\right]}^{-}$$

(Equation 14) $${\left[\text{FeCl}\left(\text{OH}\right)\right]}^{-}\to \text{FeCl}\left(\text{OH}\right)+e^{-}$$

(Equation 15) $$\text{FeCl}\left(\text{OH}\right)+H^{+}\to {\text{Fe}}^{2+}+H_{2}O+{\text{Cl}}^{-}$$

Although Cl^−^ does not participate in the electrode reaction, acidification promotes the local dissolution of Fe metal, thus intensifying the corrosion. Moreover, Cl^−^ would affect the stress corrosion cracking (SCC) of 13Cr stainless steel and increase the risk of material failure, and SCC sensitivity decreased first and then increased with the increase of Cl^−^ concentration.^94^

The general corrosion rate of carbon steel is influenced by the Cl^−^ content and exposure time. The corrosion rate tends to decrease with longer soaking time, while Cl^−^ content shows an increase in the total penetration rate of carbon steel.^18^^,^^95^^,^^96^ While in the process of coating protection, the presence of enriched chloride ions at the coating/substrate interface can compromise the effectiveness of the coating, as it can catalyze the corrosion process and promote the dissolution of the carbon steel substrate through a catalytic mechanism.^91^ In Figure 9F, the pitting depth and localized corrosion rate of the substrate in H_2_S-containing environments are quantified.

In summary, the role of chloride ions in CO_2_ corrosion is complex and often elevates the risk of corrosion. Therefore, in practical applications, it is advisable to choose alloy materials with higher resistance to chloride ion corrosion, utilize chloride ion inhibitors, or optimize coatings to mitigate the impact of chloride ions on corrosion and thereby protect equipment and pipelines.

### Other factors

Apart from the aforementioned factors, there are additional factors that can also influence CO_2_ corrosion. For example, the existence of oil can change the structure and chemical composition of the corrosion product film, thus playing a certain role in corrosion inhibition. In addition, the addition of Ca^2+^ promoted the dissolution of scale and reduced the corrosion resistance of steel to CO_2_, the mechanical properties, and wear resistance.^97^ Moreover, Kahyarian et al.^98^ found that CO_2_ and/or its related carbonate species are directly involved in the metal dissolution reaction. The presence of CO_2_ significantly influences the kinetics and the mechanism of the iron dissolution reaction. Besides, Paolinelli et al.^99^ revealed that pre-corrosion decreased the inhibitor efficiency, but its impact depended on the microstructure.

In summary, there are numerous factors that influence CO_2_ corrosion. In practice, it is crucial to comprehensively consider these factors and select appropriate corrosion control methods based on the specific application environment and metal materials. This ensures the reliability and safety of equipment and structures, reduces risks, and enhances overall reliability.

## Prevention and control approaches to carbon dioxide corrosion

Currently, the commonly used methods for corrosion protection include the use of corrosion inhibitors, coatings, and alloys. It is worth noting that inhibitors, owing to their remarkable effectiveness and cost-effectiveness, are currently the primary focus of research in this field.

### Corrosion inhibitor

**Figure 10:**
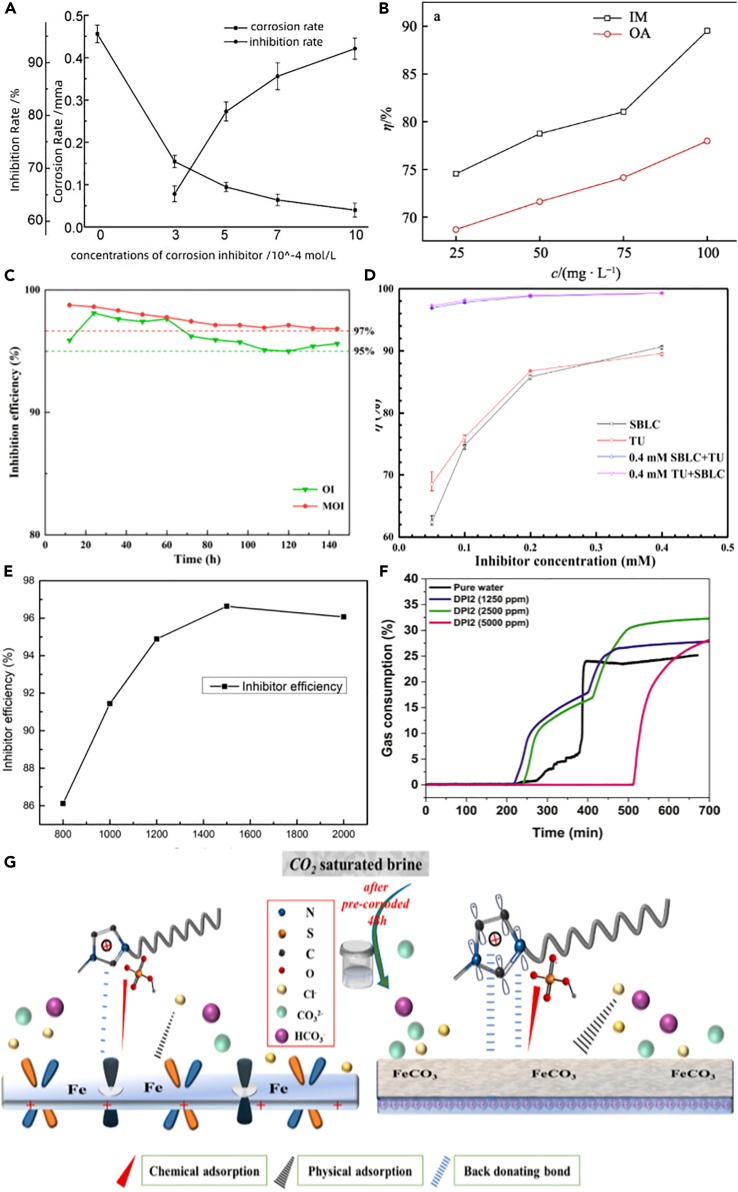
Effect diagram of corrosion inhibitor (A) Corrosion rate and inhibition rate.^101^ (B) Corrosion inhibition efficiency.^102^ (C) Inhibition efficiency. (D) Comparison of inhibition efficiency. (E) Concentration with inhibitor efficiency. (F) Gas consumption ratio curves. (G) Corrosion inhibition mechanism. Figures reproduced from: (C) (Zheng et al.^100^) Copyright 2022, Elsevier; (D) (Zhang et al.^103^) Copyright 2021, Elsevier; (E) (Zhuoke et al.^104^) Copyright 2021, BMC; (F) (Farhadian et al.^105^) Copyright 2023, Elsevier; (G) (Guo et al.^106^) Copyright 2022, Elsevier.

**Figure 11:**
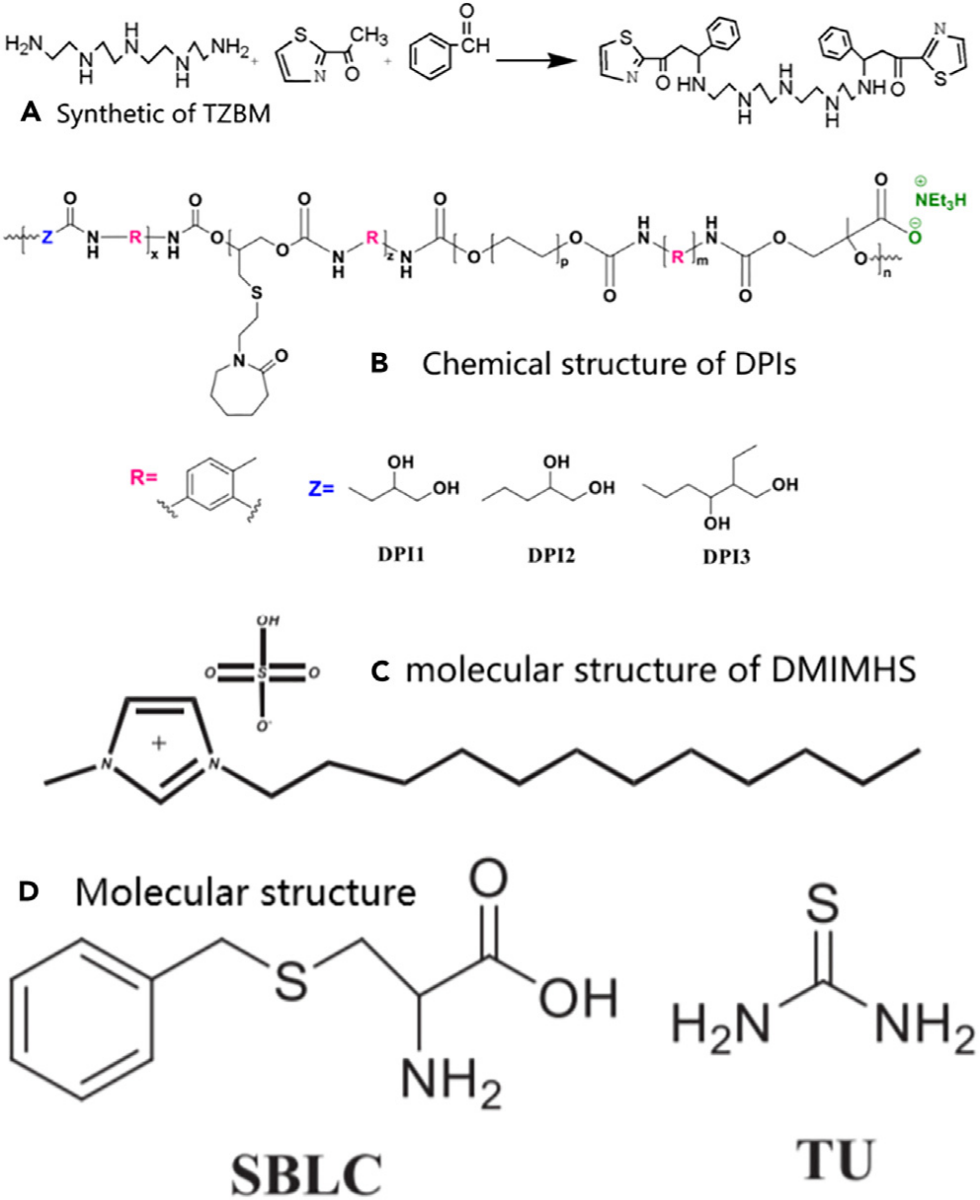
Molecular structure diagram (A) Synthetic of TZBM. (B) Chemical structure of DPls. (C) Molecular structure of DMIMHS. (D) Molecular structures of SBLC and TU. Figures reproduced from: (A) (Zhuoke et al.^104^) Copyright 2021, BMC; (B) (Farhadian et al.^105^) Copyright 2023, Elsevier; (C) (Guo et al.^106^) Copyright 2022, Elsevier; (D) (Zhang et al.^103^) Copyright 2021, Elsevier.

Corrosion inhibitor is the most commonly used method to inhibit corrosion. The mechanisms can be summarized as electrochemical mechanism and physical-chemical mechanism. The former is based on the corrosion electrochemical process of a certain step that is blocked and slows down the corrosion rate. The latter is based on the metal surface to produce adsorption or into the film and slows down the corrosion rate, which is widely employed in the protection of metallic materials. For example, Zheng et al.^100^ synthesized two imidazoline (IM) derivatives oleic imidazoline (OI) and Mercapto-oleic imidazoline (MOI) with high efficiency and confirmed that the inhibition efficiency of MOI was as high as 95.58% at 20 ppm as shown in Figure 10C. Both inhibitors are attached to the metal surface through chemisorption, while the inhibition efficiency of MOI is better than that of OI because sulfhydryl groups in MOI can be used as powerful adsorption sites, which effectively improves the adsorption capacity of MOI. Coincidentally, Li et al.^104^ obtained a bimannich-based TZBM containing a thiazole ring by synthesizing Mannich base, which effectively inhibits the corrosion of containing Cl^−^+H_2_S + CO_2_ environment as illustrated in Figure 10E. Figure 11A represent the synthetic of TZBM. The inhibitor molecules adsorbed on the surface block the corrosive medium. The corrosion inhibitor polyethylene glycol-2 oleamide functions on the similar principle.^107^

Abdolreza et al.^105^ developed the newly synthesized dual-purpose inhibitors (DPIs). Figure 11B display the chemical structure of DPIs. As exhibited in Figure 10F, DPI_2_ exhibited the highest inhibition activity on the nucleation step of hydrate crystals by delaying their formation. The strong adsorption of DPI_2_ on the metal surface makes the protective layer formed to protect the metal from corrosion. Furthermore, DPI_2_ exhibits a high level of adsorption on the metal surface, which allows for the formation of a protective layer on the mild steel.

Guo et al.^106^ used an imidazolium-based ionic liquid, 1-dodecyl-3-methyl imidazolium hydrogen sulfate (DMIMHS), as corrosion inhibitor and tested its performance. Figure 11C shows the molecular structure of DMIMHS. The addition of DMIMHS significantly improved the inhibitive properties for pre-corroded carbon steel. The proposed adsorption mechanism of DMIMHS on Fe and FeCO_3_ surfaces is illustrated in Figure 10G.

Combining different types of inhibitors, namely hybrid inhibitors, has been found to enhance their inhibitory effectiveness compared to using a single inhibitor. Furthermore, employing electrochemical methods provides a deeper understanding of the mechanisms involved in CO_2_ corrosion. This, in turn, facilitates the development of more precise and effective inhibitors. This method is instrumental for researchers in gaining insights into the corrosion processes and improving the performance of inhibitors. For instance, Wei et al.^101^ used linoleic acid and tetraethylenepentamine (TEP) as reactive monomers to synthesize a highly effective IM inhibitor (GIM). The results in Figure 10A show that the addition of GIM corrosion inhibitor reduces the corrosion rate of J55 steel sheet by increasing the charge transfer resistance on the metal substrate surface, thus reducing the corrosion current density, effectively slowing down the electrochemical reaction on the metal surface. The IM inhibitor was completely protonated under the condition of CO_2_ saturation of 3 wt %NaCl (pH∼4.1), which increased the solubility/dispersibility of the inhibitor in brine and enhanced the molecular adsorption ability of positively charged IM on metal surface.^108^

The combination of different types of inhibitors, known as hybrid inhibitors, has garnered attention. This approach enhances the effectiveness of inhibitors and makes them adaptable to various environmental conditions. Liu et al.^102^ studied the synergistic corrosion inhibition effect of IM and oleic acid (OA) on N80 steel in formation of water containing CO_2_-saturated oil field. As shown in Figure 10B, when IM and OA were added separately, the corrosion inhibition efficiency was 82.89% and 78.51%, respectively, at the mass concentration of 100 mg/L. The corrosion inhibition efficiency was increased to 98.07% after the mixture ratio of IM-OA was 25:75. The structure of the mixed film is changed after the two compounds are mixed, which makes the inhibitor film more compact, so as to achieve better corrosion inhibition effect.

Zhang et al.^103^ studied the synergistic inhibition of S-benzyl-L-cysteine (SBLC) and thiourea (TU). What can be seen in Figure 10D is that the inhibitory effect of the mix inhibitor is significantly higher than that of individual SBLC or TU at the same concentration, owing to the intermolecular interaction between SBLC and TU resulting in the formation of a denser adsorption film on the surface of carbon steel, which showed significant synergistic inhibition. Figure 11D shows the molecular structures of SBLC and TU.

In recent years, out of consideration for the environment, there has been a significant focus on environmentally friendly and green CO_2_ corrosion inhibitors. For example, Berdimurodov et al.^109^ put forward a point that using carbon dots (CDs) in corrosion protection is an ecological and environmentally efficient method because CDs have good water solubility, biocompatibility, low toxicity, excellent antibacterial properties, chemical stability, high thermal activity, and nonflammability. The lone electron pairs promote CDs to become efficient corrosion inhibitors. It is hoped that, in the near future, CDs can play an important role in corrosion inhibitors, for it is more effective at low concentrations and easy to synthesize.

Indeed, CO_2_ corrosion inhibitors offer several advantages, including environment friendly, cost-effectiveness, and compatibility. By comparison, CO_2_ corrosion inhibitors do have certain disadvantages, including limited effectiveness, application challenges, and dependency on concentration. When selecting and implementing CO_2_ corrosion inhibitors for corrosion protection in specific applications, it is important to consider various factors, such as metal type, performance requirements, cost considerations, and environmental impact.

Above all, the forefront of corrosion inhibitor research is centered on the exploration of novel and environmentally friendly inhibitors, the utilization of hybrid inhibitors, and delving deeper into electrochemical investigations. These advancements hold the promise of enhancing control over CO_2_ corrosion, reducing equipment degradation, and mitigating adverse environmental impacts.

### Coating protection

Coatings offer several advantages, including excellent corrosion resistance, long-term protection, and adaptability. Considering these advantages, coatings play a crucial role in safeguarding structures and equipment against corrosion. The underlying mechanisms primarily involve isolation and shielding principles. The former is achieved by forming an isolating layer that separates the metal surface from CO_2_ gas, thus reducing the reactivity between the corrosion medium and the metal. For example, Peng et al.^110^ used the water-based modified epoxy resin as the anti-corrosion material to improve the anti-corrosion performance of oil well cement. Compared with cement stone without modified epoxy resin, the modified epoxy resin mainly improved the corrosion resistance of cement paste by filling pores to improve the compactness of cement paste structure and forming a three-dimensional network polymer film to isolate acidic media.

The latter provides a sacrificial shielding layer that is more susceptible to damage than the metal itself, confining corrosion damage to the coating rather than the metal surface, similar to the principle of sacrificial anodes. Commonly used coatings include nickel-phosphorus (Ni-P) coatings, zinc coatings, polymer coatings, and organic coatings. Achieving optimal protective performance often depends on the selection of coating materials, which is influenced by factors such as the application’s environmental conditions and the type of metal.

**Figure 12:**
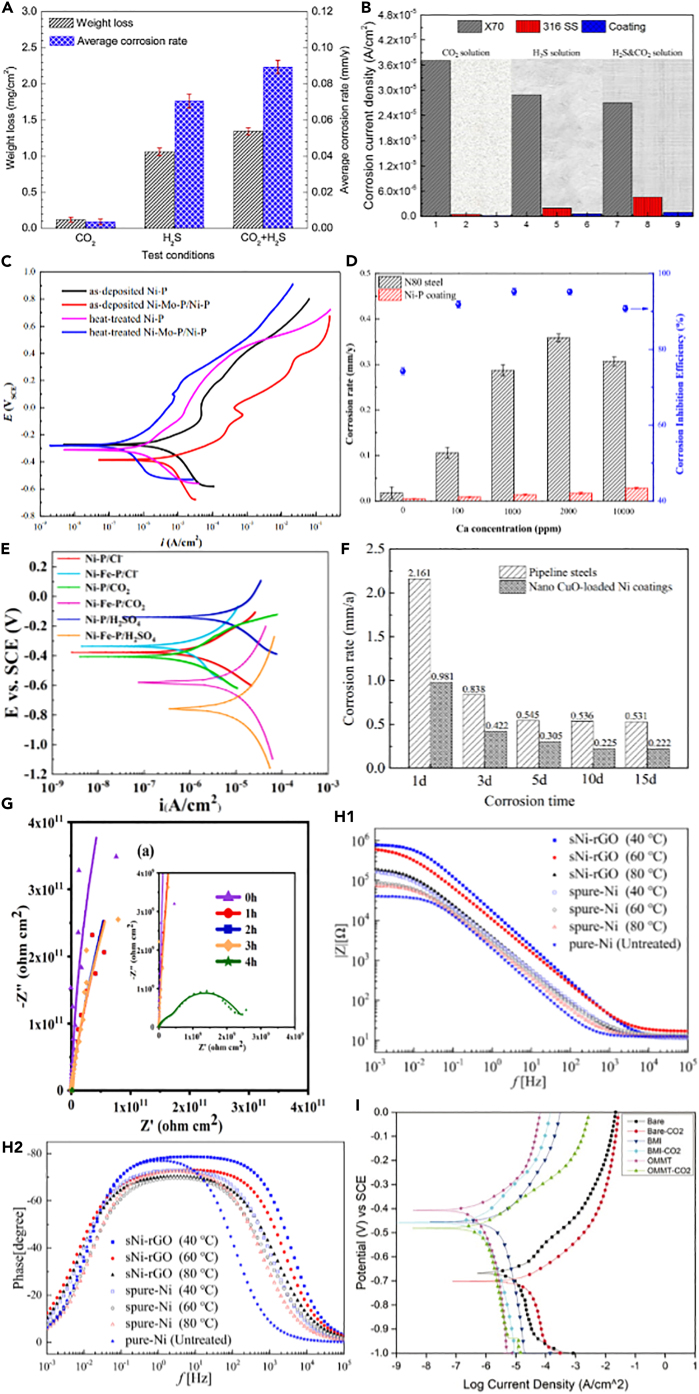
Coating protection effect (A) Weight loss and average corrosion rate. (B) Corrosion current density. (C) Potentiodynamic polarization curves. (D) Corrosion rates and corrosion inhibition efficiencies. (E) Tafel curves. (F) Corrosion rate and time. (G) Nyquist plots. (H1-H2) Bode diagram. (I) Potentiodynamic polarization curves. Figures reproduced from: (A) (Sun et al.^91^) Copyright 2019, Elsevier; (B) (Wang et al.^111^) Copyright 2016, Elsevier; (C) (Li et al.^112^) Copyright 2020, Elsevier; (D) (Li et al.^113^) Copyright 2021, Elsevier; (E) (Zhang et al.^114^) Copyright 2022, Elsevier; (F) (Zhu et al.^115^) Copyright 2023, Elsevier; (G) (Luo et al.^116^) Copyright 2022, Elsevier; (H) (Wang et al.^117^) Copyright 2021, John Wiley and Sons; (I) (Al Shenawa et al.^118^) Copyright 2021, Taylor & Francis.

For Ni-P coating, it showed good corrosion resistance in CO_2_ environment, especially in the presence of high chloride ions. But in H_2_S and CO_2_-H_2_S environment as shown in Figure 12A, the coexistence of them led to CO_2_ enhancing H_2_S corrosion effect, and the synergistic effect accelerated the degradation of the coating.^91^ The addition of H_2_S accelerates the diffusion process of electrolyte/coating interface and promotes the penetration of electrolyte through the coating, resulting in serious local corrosion and coating peeling.^119^ Nevertheless, as displayed in Figure 12B, the morphology of Ni-Cr-Mo coating has no change after corrosion in the simulated solution environment of CO_2_, H_2_S, and their mixture, with higher corrosion resistance.^111^ The Ni-Cr-Mo coating has low cost and excellent performance. It is feasible to apply it to the corrosion protection of petroleum industry.

To enhance the corrosion resistance and improve the passivation properties of the coating, Li et al.^112^ applied an electroless Ni-Mo-P/Ni-P composite coating on N80 carbon steel, which involved adding molybdenum and undergoing heat treatment. As depicted in Figure 12C, heat-treated coatings exhibit lower corrosion activity, as evidenced by the leftward shift of the curves (indicating smaller current densities) compared to as-deposited coatings. In addition, the concentration of Ca^2+^ has a significant impact on the corrosion of N80 steel, whereas the Ni-P coating shows excellent corrosion resistance and is hardly affected by Ca^2+^, as demonstrated in Figure 12D.^113^ Furthermore, the addition of iron to the Ni-P coating can improve its flatness, increase grain size, induce crystallization, and facilitate the transformation of Ni(OH)_2_ to NiO in the passivation film, as exhibited in Figure 12E.^114^ However, it is not recommended for use in highly acidic environments. This is because the iron within Ni-Fe-P coatings can undergo dissolution due to the presence of H^+^ ions, resulting in the deterioration of the coating’s structure and eventual detachment from the substrate surface.

The addition of other elements or compounds to Ni-P coatings is also a promising research direction. This approach has the potential to enhance their performance, reduce adverse environmental effects, and broaden their applicability through alloying. For example, Zhu et al.^115^ electroplated Ni coating on pipeline steel surface and incorporated CuO nanoparticles to impart antibacterial functionality. As shown in Figure 12F, the corrosion rate was decreased due to the reduced probability of corrosion reaction by the presence of nano-CuO and its antibacterial effect against sulfate-reducing bacteria. This research provides new insights for addressing microbial corrosion issues in oil and gas pipelines.

Graphene oxide (GO) holds immense potential as an efficient coating, which is typically combined with other materials, such as polymers or nanoparticles, to create composite inhibitors. This further enhances the performance in mitigating CO_2_ corrosion and provides long-lasting protection. For instance, Luo et al.^116^ modified GO nanosheets with the photocatalytic heterostructure TiO_2_-ZnO and fabricated TiO_2_-ZnO-GO (T-Z-G) ternary nanofillers to block the intrusion of corrosive media through the layer structure of GO. As illustrated in Figure 12G, the coating has excellent anti-fouling, anti-corrosion, anti-corrosion, and barrier properties and can be applied to CO_2_ storage and transportation protection in CCUS process.

Oppong Boakye et al.^120^ investigated polymer coating modified with GO and duplex electroless Ni-P with polytetrafluroethylene (PTFE) coating for the infrared corrosion behavior. At 120°C, the polymer coating with added GO nanosheets displayed reduced wetting ability and effectively suppressed the corrosion effects of the substrate. However, none of the coatings provided protection at 250°C in the H_2_S/CO_2_ environment. Therefore, when applying this coating, it is important to consider practical environmental temperature, in order to prevent coating failure. But Wang et al.^117^ fabricated a novel superhydrophobic nickel-reduced graphene oxide (sNi-rGO) coating on N80 steel substrate. As shown in Figure 12H, the sNi-rGO coating has excellent corrosion resistance in simulated well fluid. The coating still maintained superhydrophobicity and corrosion resistance under the condition of high-temperature and high-pressure CO_2_.

Research into the incorporation of ceramic materials within coatings remains an active and dynamic field. The fundamental concept involves the amalgamation of ceramic particles or ceramic materials within a coating to enhance its resistance. For example, Al Shenawa et al.^118^ analyzed the effect of salt water corrosion in filled and unfilled ceramic filled polymer coatings before and after supercritical CO_2_ exposure. As revealed in Figure 12I, after exposure to CO_2_, the filled and unfilled systems had similar corrosion properties, while the filled coating had better wear resistance.

Coating and cathodic protection are effective measures to prevent pipeline corrosion. Although coating can provide excellent corrosion protection, it can degrade over time or become ineffective due to factors such as water absorption, blistering, and damage. Thus, it is crucial to carefully select appropriate coating materials, surface treatment processes, optimal working temperatures, suitable coating thicknesses, and other relevant factors based on the specific situation to achieve the best possible protection. Furthermore, regular inspection and maintenance of coatings, corrosion rate monitoring, and training for operational and maintenance personnel are also indispensable. A comprehensive consideration of these points will aid in reducing the risks associated with corrosion, ultimately extending the lifespan of pipelines.

### Alloy protection

**Figure 13:**
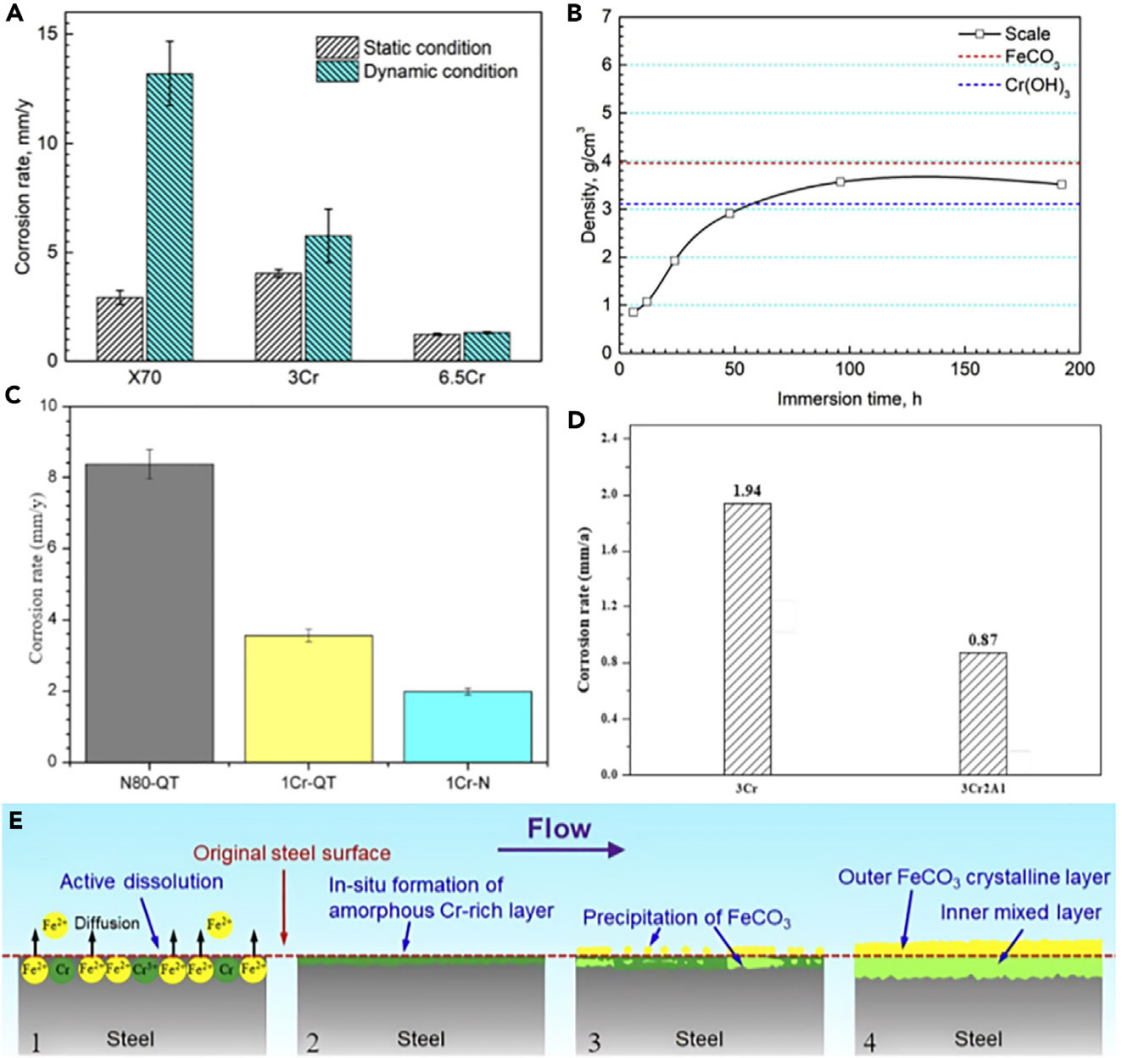
Alloy protection efficiency (A) Corrosion rates. (B) Average density. (C) Corrosion rates comparison. (D) Corrosion rate. (E) Schematic formation mechanism. Figures reproduced from (A, B, and E) (Wei et al.^121^) Copyright 2019, Elsevier; (C) (Wu et al.^122^) Copyright 2013, Elsevier; (D) (Gao et al.^123^) Copyright 2022, Emerald Publishing Limited.

Alloy protection refers to a kind of protection means through element control, lattice control, and matching with metal production process. Selecting appropriate corrosion-resistant materials can also effectively reduce the corrosion risk of CO_2_ and improve the stability of equipment. For example, the addition of aluminum in low-Cr steel helped to form a dense corrosion product layer, greatly reducing the local corrosion sensitivity (Figure 13D).^123^ Figure 13C indicates that the corrosion resistance of carbon steel such as N80 can be improved by adding small amounts of alloy elements.^122^

The enhancement of corrosion resistance in Cr (chromium) alloys through alloy design and optimization constitutes a pivotal component of alloy protection strategies. However, under static conditions, the incorporation of a small amount of Cr cannot facilitate the formation of a robust Cr(OH)_3_ protective layer.^121^ As shown in Figures 13A and 13B, 6.5Cr steel has good corrosion resistance because the corrosion products of 6.5Cr under dynamic and static conditions are single-layer chromium-rich layer composed of amorphous FeCO_3_ and Cr(OH)_3_. The good local corrosion resistance of Cr-containing steel is attributed to the *in situ* formation of the initial amorphous Cr-rich layer. Figure 13E displayed schematic diagram of the formation mechanism of corrosion product scale on 3Cr steel in dynamic s-CO_2_-saturated aqueous phase.

Kai et al.^124^ studied the effect of surface finishing on the oxidation behavior of a Fe-21Cr-32Ni alloy in s-CO_2_. The high Cr diffusion led to the transition from internal to external oxidation by favoring the formation of a thin/protective Cr-rich oxide layer. Furthermore, it reduced the permeability of carbon into the oxides, thus improving the oxidation and carburization resistance of the ground specimen. In the context of high-purity research-grade CO_2_, the corrosion resistance of the Ni-based alloy Haynes 230 proved to be excellent.^125^ This superiority can be attributed to the formation of a remarkably thin, consistently uniform, and protective layer enriched with chromium oxide on its surface.

The effectiveness of alloy protection against CO_2_ corrosion is significant; however, its high cost and complex processing are important factors that limit its development. Additionally, the maintenance and repair of the alloy are typically more intricate compared to conventional materials, and one must also consider compatibility when it comes into contact with other metals. Therefore, the research focus in the future should be on the development of low-cost and easy-to-process alloys to enable large-scale application. Simultaneously, there is a need for a focus on material modeling and simulation calculations to predict the corrosion behavior of the alloy under various conditions, thereby expediting the development of high-performance environmentally friendly alloy materials.

## Conclusion and outlook

CCUS is an effective approach to reducing carbon emissions, but the issue of CO_2_ corrosion cannot be overlooked. This review elucidates CO_2_ corrosion problems, including its mechanism and influencing factors, and summarizes anti-corrosion methods. It particularly underscores the distinctive advantages of inhibitors.

Unfortunately, the field of corrosion-resistant materials still faces challenges, such as high cost and difficulty in processing. Although it can efficiently mitigate corrosion, further research and improvement are required to ensure its economic feasibility. While the cost of coating protection is relatively low, there are still some areas that require improvement, such as long-term durability, applicability, and sustainability.

Another promising direction is developing novel green inhibitors. Inhibitor is currently one of the most commonly applied methods, offering good corrosion resistance and superior economy. However, improper discharge may result in detrimental environmental consequences due to its corrosive nature. Currently, nitrogen-based compounds like IMs serve as effective inhibitors with good anti-corrosion performance, and they represent an essential research direction for the future. Moreover, the high adaptability and effectiveness demonstrated by mixed inhibitors have sparked considerable research interest and represent one of the key areas for future investigations. Developing high-performance and environmentally friendly corrosion inhibitors to enhance stability and optimize usage methods is a top priority.

In the future, the authors recommend CO_2_ corrosion protection research could focus on the following areas.

Although significant progress has been made in corrosion inhibitor research worldwide, the overall progress is still relatively limited. The development of green corrosion inhibitors is crucial for future research. The focus should be on preparing environmentally friendly and efficient corrosion inhibitors while explaining their compliance with green standards and corrosion inhibition mechanisms. Computer-based molecular simulation methods have been applied in corrosion inhibitor research, providing a useful tool for future molecular design. As oil field production technology and the environment continue to evolve, the stability and durability of corrosion inhibitor performance are challenged under harsh conditions; specific research on the application of these coatings in oil field production technology and actual environments should be carried out to determine their effectiveness in practical situations. Coatings gradually soften with increasing temperatures, so future research can focus on developing coatings with high temperature and pressure resistance, acid resistance, and compactness to improve permeability resistance.
